# Consequences of CRISPR-Cas9-Mediated CFTR Knockout in Human Macrophages

**DOI:** 10.3389/fimmu.2020.01871

**Published:** 2020-08-18

**Authors:** Shuzhong Zhang, Chandra L. Shrestha, Benjamin L. Wisniewski, Hanh Pham, Xucheng Hou, Wenqing Li, Yizhou Dong, Benjamin T. Kopp

**Affiliations:** ^1^Center for Microbial Pathogenesis, The Abigail Wexner Research Institute at Nationwide Children's Hospital, Columbus, OH, United States; ^2^Division of Pulmonary Medicine, Nationwide Children's Hospital, Columbus, OH, United States; ^3^Pharmaceutics and Pharmacology, The Ohio State University, Columbus, OH, United States

**Keywords:** cystic fibrosis, macrophage, bacteria, CRISPR, CFTR

## Abstract

Macrophage dysfunction is fundamentally related to altered immunity in cystic fibrosis (CF). How genetic deficits in the cystic fibrosis transmembrane conductance regulator (CFTR) lead to these defects remains unknown. Rapid advances in genomic editing such as the clustered regularly interspaced short palindromic repeats associated protein 9 (CRISPR/Cas9) system provide new tools for scientific study. We aimed to create a stable CFTR knockout (KO) in human macrophages in order to study how CFTR regulates macrophage function. Peripheral blood monocytes were isolated from non-CF healthy volunteers and differentiated into monocyte-derived macrophages (MDMs). MDMs were transfected with a CRISPR Cas9 CFTR KO plasmid. CFTR KO efficiency was verified and macrophage halide efflux, phagocytosis, oxidative burst, apoptosis, and cytokine functional assays were performed. CFTR KO in human MDMs was efficient and stable after puromycin selection. CFTR KO was confirmed by CFTR mRNA and protein expression. CFTR function was abolished in CFTR KO MDMs. CFTR KO recapitulated known defects in human CF MDM (CFTR class I/II variants) dysfunction including (1) increased apoptosis, (2) decreased phagocytosis, (3) reduced oxidative burst, and (4) increased bacterial load. Activation of the oxidative burst via nicotinamide adenine dinucleotide phosphate (NADPH) oxidase assembly was diminished in CFTR KO MDMs (decreased phosphorylated p47^phox^). Cytokine production was unchanged or decreased in response to infection in CFTR KO MDMs. In conclusion, we developed a primary human macrophage CFTR KO system. CFTR KO mimics most pathology observed in macrophages obtained from persons with CF, which suggests that many aspects of CF macrophage dysfunction are CFTR-dependent and not just reflective of the CF inflammatory milieu.

## Introduction

Cystic fibrosis (CF) is a multi-system disorder characterized by chronic polymicrobial pulmonary infections that lead to progressive morbidity (1). CF pathophysiology results from variants in the cystic fibrosis transmembrane conductance regulator (CFTR) gene, which lead to absent or dysfunctional CFTR protein expression. CFTR is present in epithelial-lined organs throughout the body, as well as in immune cells including macrophages (2, 3). While the persistence of pathogens in CF is multi-factorial, recent evidence suggest that alterations in the innate immune system are critically related to both acute and chronic infections in CF. (2, 4–17). However, the overall relationship between CFTR deficits and immune function remains poorly defined.

Macrophages have an important role in CF immune dysfunction due to multiple defects including reduced phagocytosis (2, 18–20) and a defective oxidative burst (4) among others (21, 22). How genetic alterations in CFTR contribute to these defects remains unknown. Recent advances in genomic editing such as the clustered regularly interspaced short palindromic repeats associated protein 9 (CRISPR/Cas9) system have provided unique tools for scientific study. However, there are limited strategies to allow reliable and efficient gene editing in human macrophages due to their terminally differentiated state. Although new viral vectors, polymers, or lipid chemical complexes can improve genetic editing approaches, many can induce untoward immune responses (23, 24). Thus, we aimed to create a stable CFTR knockout (KO) in human macrophages to study how CFTR regulates macrophage function. We hypothesized that macrophage effector functions are directly dependent on CFTR and therefore, a CFTR KO would reflect the functional macrophage deficits observed in primary human CF macrophages with CFTR (class I/II) variants that result in little or no CFTR protein expression.

## Materials and Methods

### Human Subjects

Thirty-two healthy non-CF, non-smoking, adult controls were recruited with approval by the Institutional Review Board of Nationwide Children's Hospital (IRB16-01020). All participants provided written informed consent. Male (40%) and females (60%) were recruited, with an average age of 36 years (range 22–66 years). Research procedures were done according to local and international guidelines and regulations for human research. Subject information was stored in a REDCap database.

### Macrophage Isolation

Blood samples in Ethylenediaminetetraacetic acid (EDTA) tubes (Becton Dickinson 367861) were obtained from non-CF healthy controls per prior methods (2). In brief, monocytes were isolated from whole blood using Lymphocyte Separation Medium (Corning, 25-072-CV). Isolated monocytes were cultured in Roswell Park Memorial Institute (RPMI) media (Gibco, 22400-089) plus 20% human AB serum (Corning, 35-060-Cl) and differentiated for 5 days at 37°C into MDMs as previously described (6, 25). MDM purity and integrity was confirmed by microscopy and flow cytometry. MDMs were plated in a monolayer culture with fresh RPMI, rested for 4 days, and then infected with a bacterial multiplicity of infection (MOI) ranging from 2 to 50.

### Bacterial Strains and Colony Forming Unit Assay

Macrophage infections occurred with multi-drug resistant CF clinical isolates. The *Pseudomonas aeruginosa* isolate was obtained from a CF patient's sputum. The *Burkholderia cenocepacia* k56-2 strain is an isolate of the epidemic clinical strain from the Edinburgh-Toronto (ET12) lineage (26). Bacteria were grown in Luria-Bertani (LB) media for 24 h prior to use. Colony forming unit (CFU) analysis was performed as previously described (8). Quantification of bacteria was performed by plating serial dilutions on LB agar plates.

### CRISPR-Cas9 CFTR Knockout

We optimized our prior brief description of CRISPR-Cas9 CFTR KO (2). Monocytes were differentiated for 5 days into MDMs and then 10 million MDMs were suspended in 0.85 ml of antibiotic-free RPMI medium with 10% AB serum and kept on ice for use. A mixture of N-[1-(2,3-Dioleoyloxy)propyl]-N,N,N-trimethylammonium methyl-sulfate (DOTAP, Roche 11202375001) liposomal transfection reagent and CFTR double nickase plasmid (NIC, Santa Cruz sc-400653) was then prepared according to the manufacturer's instructions. Briefly, a plasmid solution (A) was prepared by diluting 2.5 μg of plasmid to a final volume of 50 μl in 4-(2-hydroxyethyl)-1-piperazineethanesulfonic acid (HEPES)-buffered saline pH 7.4 (HBS, Bioworld 40820012) in a sterile polystyrene tube. A transfection solution (B) was prepared by mixing 50 μl DOTAP with HBS buffer to a final volume of 100 μl. Solution A was added dropwise directly to solution B and mixed gently by pipetting and incubated for 15 min at room temperature. The DOTAP/plasmid mixture was added directly to the MDMs and gently mixed by repeated pipetting. The MDM/plasmid suspension was transferred to a sterile standard vial (Savilex 200-015-12) and incubated for 14 h at 37°C. MDMs were then plated with new media and incubated for another 20 h. Finally, the supernatant was aspirated and replaced with fresh media containing puromycin at 2 μg/ml for additional 24 h before harvesting transfected MDMs for protein or RNA extraction. RNA was extracted from MDMs using the Total RNA Purification Kit (Norgen Biotek, 17200). Semi-quantitative reverse transcription polymerase chain reaction (qRT-PCR) was then performed by synthesizing complementary DNA (cDNA) by reverse transcriptase (Santa Cruz, sc43500) and then using nested PCR via two pairs of CFTR primer (CFTR (h)-PR, Santa Cruz, sc35054-PR) for determining CFTR transcription level. 18S rRNA (Forward: 5′-GGTGAAATTCTTGGACCGGC-3′; Reverse: 5′-GACTTTGGTTTCCCGGAAGC-3′) was used as an internal control.

### Immunoblotting

MDM supernatants were removed post treatment and the cells were washed twice with phosphate buffered saline (PBS, Corning 21030-CV). MDMs were lysed in lysis buffer (10 mM HEPES, 5 mM MgCl_2_, 1 mM EGTA, 140 mM KCL,1% NP-40) with a protease inhibitor (Roche Applied Science, 10-519-978-001). Then 30 μg of protein was denatured in Laemmli sample buffer for 10 min at 95°C, and then separated by SDS-PAGE and transferred onto polyvinylidene difluoride membranes. Membranes were immunoblotted for β-actin (Cell Signaling, 8H10D10), anti-CFTR (Abcam CF3 2784), anti-CFTR (596) obtained from the CFTR Antibody Distribution Program https://www.cff.org/Research/Researcher-Resources/Tools-and-Resources/CFTR-Antibodies-Distribution-Program/, phospho-p47^phox^ (donated by Jamel El-Benna), and total p47phox (Life Technologies, A16636). Protein bands were detected with HRP-conjugated secondary antibodies and visualized using enhanced chemiluminescence reagents (Life Sciences, RPN2106). Membrane and cytosolic fractionations were prepared via manufacturer kit instructions (Thermo Fisher, 78840). Bands were quantified by densitometric scanning of the films and analyzed using ImageJ.

### CFTR-Mediated Halide Efflux

Non-CF and CFTR KO MDMs were plated in a 96-well plate at 1 × 10^6^/100 μl/well and rested for 48–72 h prior to use. Media was then removed and cells were washed with efflux solution (mM):135 NaNO_3_, 1 CaSO4, 1 MgSO4, 2.4 K2HPO4, 0.6 KH2PO4, 10 HEPES, and 10 Glucose. A fluorescent indicator of intracellular Cl^−^ (N-Ethoxycarbonylmethyl-6-Methoxyquinolinium Bromide [MQAE], Thermofisher E3101) was loaded with a hypotonic buffer (0.56 ml 36 mM MQAE stock with 1 ml of filtered deionised water and 0.44 ml of efflux solution) for 5 min at 37°C in the dark. MDMs were then washed with efflux solution to remove extraneous MQAE and incubated in 100 μl/well of warmed halide efflux solution for 5 min at 37°C. The maximum fluorescence was then recorded on a plate reader. The halide efflux solution was removed and intracellular MQAE was quenched with warmed NaI (Sigma, 409286) buffer (135 mM NaI in efflux buffer, 100 μl/well) at 15 min at 37°C. NaI buffer was removed and halide efflux solution added back for 5 min and basal fluorescence measured. Cells were then incubated with forskolin (20 μM, Sigma F6886) and 3-isobutyl-1-methylxanthine (IBMX, 100 μM, Sigma 15869) or CFTR inhibitor 172 (CFTRinh172, 10 μM, Simga C2992) at 37°C and fluorescence measured every 5 min for 30 min. The minimum fluorescence was obtained by exposing cells to the quenching buffer (150 mM KSCN, 5 μM valinomycin in NaI buffer) for 30 min at 37°C. CFTR-dependent chloride efflux was calculated as maximum fluorescence after forskolin stimulation.

### Apoptosis Assay

Apoptosis was performed per prior methods (2). Briefly, MDMs were plated at a density of 1 × 10^6^/ml in 12 well plates. Apoptosis was measured by flow cytometry and fluorescence-activated cell sorting (FACS) analysis using APC Annexin V (Biolegend, 640920) and propidium iodide (BioLegend 421301). MDMs were detached by Accutase solution, collected, washed, re-suspended in 100 μl of Annexin V Binding Buffer (Biolegend 422201), and then stained with 5 μl of Annexin V and 10 μl propidium iodide for 15 min at room temperature in the dark. The percentage of viable and apoptotic cells was calculate using flow cytometry (BD LSR II Flow Cytometer).

### Phagocytosis Assay

Phagocytosis was performed per prior methods (2). Briefly, Sphero^TM^ Ultra Rainbow Fluorescent beads (bead diameter 3–3.4 μM, Spherotech Inc.) were opsonized with human serum at 37°C for 45 min and layered onto MDMs in a ratio of 50:1 (beads to cells) for 18 h, washed twice with PBS, re-suspended in Hank's balanced salt solution and then imaged with an inverted fluorescence microscope or detached by Accutase (Sigma 16964) for FACS analysis. For the bacterial phagocytosis assay, RFP-expressing *B. cenocepacia* were fixed using 4% paraformaldehyde for 30 min at room temperature. They were then washed five times with PBS and re-suspended in PBS containing 10% AB serum for 60 min at 37°C. Serum-opsonized *B. cenocepacia* were incubated with MDMs at 50:1 (bacteria to cells) for 40 min at 37°C and then washed with PBS. Last, MDMs were detached and the percent uptake of RFP-expressing *B. cenocepacia* was detected by FACS analysis.

### Reactive Oxygen Species (ROS) Assay

The oxidative burst was measured by a 2′,7′-dichlorofluorescein (DCF) assay (Life Technologies, D399) using relative fluorescent units (RFUs). MDMs were adhered to 96 well plates at 4 × 10^6^ cells/well in duplicate for 2 h, and repleted in Dulbecco's PBS with 10 mM HEPES and 1 mg/ml human serum albumin plus 0.1% glucose (DPBS-HHG). After a 30-min incubation at 37°C, 10% DCF was added to the wells for 30 min at 37°C. A phorbol 12-myristate 13-acetate (PMA) st imulus was added to MDMs and fluorescence measured at a 485 nm excitation wavelength and a 515 nm emission wavelength every 2 min for 2 h. In preliminary experiments, PMA was used at varying concentrations, with 200 μM determined to be optimal for DCF experiments to activate protein-kinase C (PKC)-mediated NADPH oxidase ROS production.

### Cytokine Production

Cytokines were measured per prior methods (2). MDM supernatants were collected at baseline or during infection with *B. cenocepacia* for 18 h, harvested, and stored at −20°C until assayed. Cytokine levels were measured using a cytometric Bead Array (CBA) Human Inflammatory cytokine kit (BD Biosciences, 551811) according to the manufacturer's instructions.

### Macrophage Polarization

MDMs were cultured in 12-well plates and exposed to an M1 polarization stimulus using *E. coli* lipopolysaccharide (LPS) (Sigma, 20 ng/ml) plus recombinant human interferon (rh-IFN)-γ (Fisher, 20 ng/ml) and into M2a using rhIL-4 (20 ng/ml) for 48 h per prior methods (2, 27). Resident cells without any stimulation were used for baseline comparison. Aliquots incubated in human Fc-γ Block (Biolegend, 422301) for 30 min on ice and then stained using phycoerythrin (PE) anti-human CD80 (Biolegend, 305208, Clone 2D10) and phycoerythrin cyanine tandem conjugate (PE/Cy7) anti-human CD11b (Biolegend, 101216, Clone M1/70) or allophycocyanine (APC) anti-human CD163 (Biolegend, 326510, Clone RM3/1) and allophycocyanin cyanine 7 tandem conjugate (APC/Cy7) anti-human CD206 (Biolegend, 321120, Clone 15-2). Next, FITC anti-human CD68 (Biolegend, 333806, Clone Y1/82A) intracellular staining was performed post-fixation and permeabilization according to the manufacture's recommendations. Stained cells were gated via viable CD11b^+^subsets and identified by CD68CD80 (M1) and CD163CD206 (M2) markers using FlowJo (Tree Star).

### Statistical Analysis

Statistical analyses were calculated with GraphPad Prism software (version 8.2). Two sample unpaired *t*-tests were used for independent sample comparisons. One-way ANOVA was used for denistometry and cytokine analysis with *post-hoc* Tukey tests. Statistical significance was defined as a *p* < 0.05. Each individual served as their own internal age- and gender-matched control during experiments when possible (KO vs. no treatment). However, due to cell recovery differences, this was not possible for all replicates and therefore paired *t*-tests were not performed.

## Results

### CFTR KO Model

**Figure 1:**
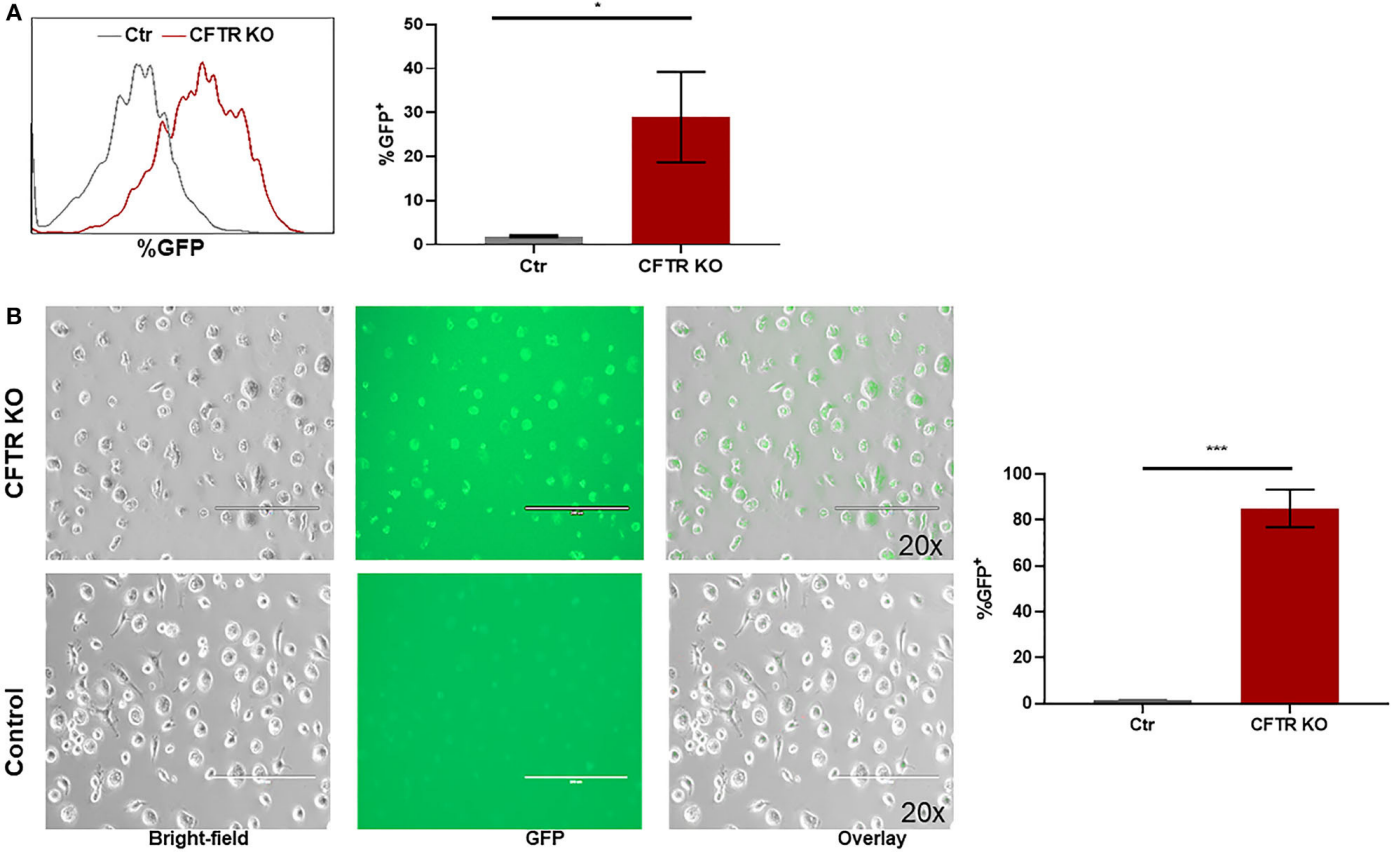
Setup of CFTR KO model. **(A)** Representative flow cytometry histogram and corresponding summary of GFP detection of CFTR KO MDMs prior to puromycin selection. *p < 0.05 via unpaired *t*-test, *n* = 3, mean ± SD. **(B)** Representative GFP fluorescence images of hMDMs transfected by CFTR Crispr Double Nickase plasmid in DOTAP with corresponding summary of GFP detection, *n* = 3. (Top) 48 h selection by puromycin. (Bottom) hMDMs in DOTAP only as control. 200 μm scale bar. Bright-field, GFP^+^, and overlay images shown in left to right order. ***p < 0.0001.

**Figure 2:**
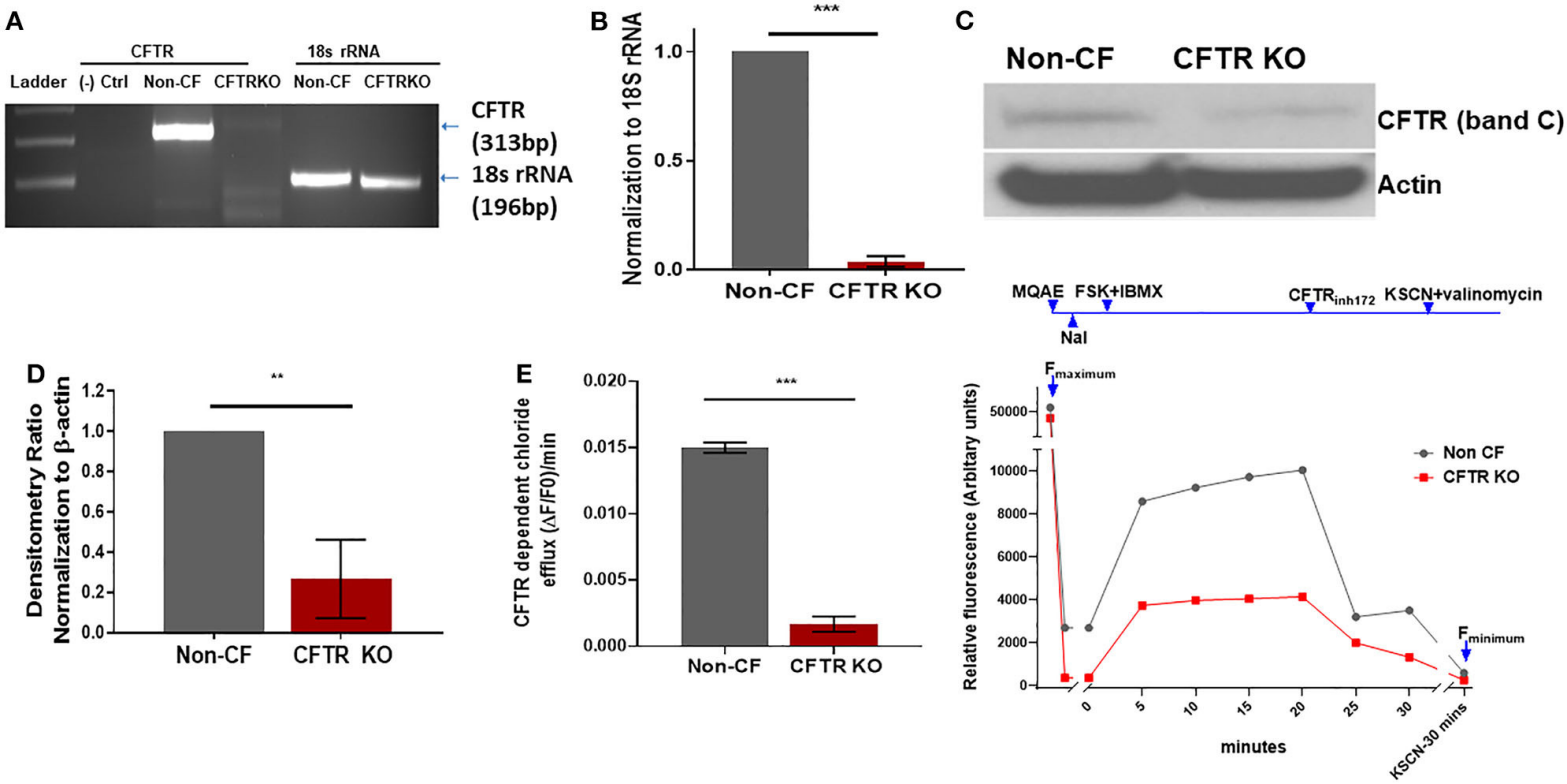
CFTR changes in CFTR KO model. **(A)** Representative semi-quantitative RT-PCR for CFTR and **(B)** corresponding quantitative summary of CFTR RT-PCR in CFTR KO and non-CF MDMs, *n* = 3, mean ± SD. **(C)** Representative CFTR western blot (Abcam CF3 2784) and **(D)** summed densitometry of CFTR KO western blots in CFTR KO and non-CF MDMs, *n* = 3, mean ± SD. **(E)** MQAE halide efflux assay of CFTR dependent chloride efflux as measure of CFTR function in non-CF and CFTR KO MDMs, *n* = 6. Maximal fluorescence after forskolin stimulation shown. Representative time course also shown with annotations for forskolin plus IBMX stimulation (FSK + IBMX), CFTR inhibition (CFTRinh172), and quenching with KSCN plus valinomycin. **p < 0.01, ***p < 0.001, via unpaired *t*-test, mean ± SD.

CFTR KO was optimized in non-CF MDMs as described in the methods. The methods section details the timing of transfection, length of incubation, puromycin selection of transfected cells, and use of DOTAP as a transfection media. We found that KO was more effective in differentiated macrophages compared to monocytes during preliminary experiments (data not shown). CFTR KO was first determined visually through identification of flow cytometry sorted GFP^+^ MDMs prior to puromycin selection. Mean initial transfection efficiency was 30% (Figure 1A). After puromycin selection, CFTR KO efficiency was visually determined at >85% (Figure 1B). To confirm CFTR KO, we measured CFTR expression via qRT-PCR. CFTR expression was significantly reduced in CFTR KO MDMs compared to control MDMs as shown in Figure 2A and summed quantification in Figure 2B. CFTR protein expression was also significantly reduced via western blot (representative blot in Figure 2C and summed densitometry in Figure 2D). All western blots can be found in (Supplementary Figure 1), which also includes an example comparison of different CFTR antibodies. We further confirmed the specificity of CFTR KO by measuring CFTR function via CFTR-dependent chloride efflux. CFTR function was abolished in CFTR KO MDMs compared to untreated vector control non-CF MDMs (Figure 2E). Combined, these results supported robust CFTR KO via our new model as measured through multiple techniques.

### CFTR KO Alters Macrophage Stability and ROS Production

**Figure 3:**
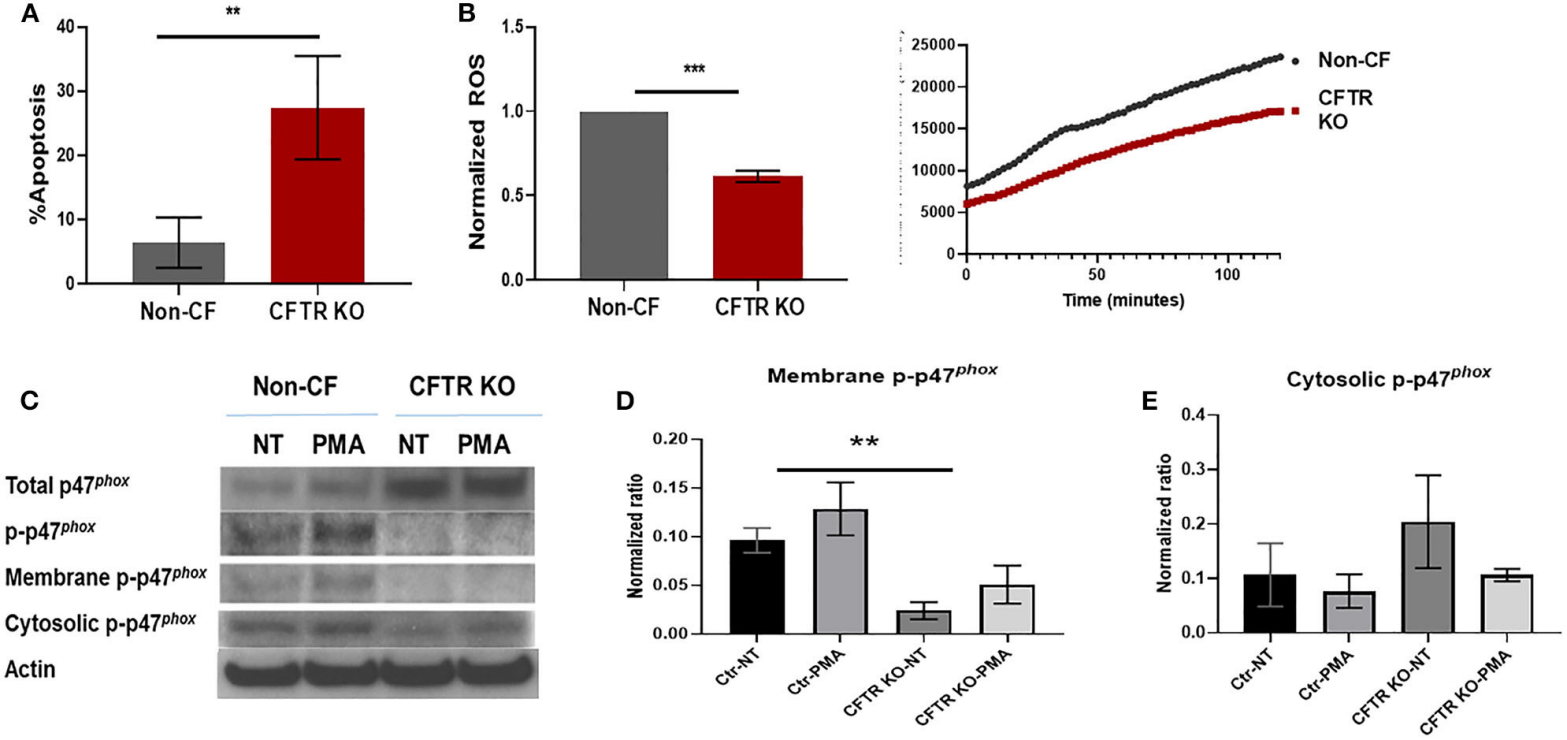
CFTR KO alters macrophage stability and ROS production. **(A)** Summed apoptosis as measured by flow cytometric detection of Annexin V in CFTR KO and non-CF MDMs, *n* = 4, mean ± SD. **(B)** Summed end-point analysis of maximum 2 h ROS production in response to PMA. ROS shown as ratio of max ROS normalized to non-CF. Representative time course of ROS production also shown. *n* = 3, mean ± SD. **(C)** Western blots of total, phosphorylated, and membrane and cytosolic fractions of phosphorylated p47^*phox*^ in non-CF and CFTR KO MDM lysates in response to no treatment (NT) or PMA exposure. Actin shown as loading control. Summed densitometry of western blots for **(D)** membrane, *n* = 3 and **(E)** cytosolic fractions of phosphorylated p47^*phox*^, *n* = 4, in non-CF and CFTR KO MDMs stimulated with PMA, mean ± SD. **p < 0.01, ***p < 0.001, via unpaired *t*-test for **(A,B)** and one-way ANOVA for **(D,E)**.

Next, we sought to determine whether previously observed deficits in human CF macrophages (2, 4) were recapitulated by CFTR KO. We measured baseline apoptosis of unstimulated non-CF and CFTR KO MDMs in culture via annexin V staining. CFTR KO MDMs had 3–4 times higher apoptosis compared to non-CF MDMs (Figure 3A), which is similar to our previous data in primary cells (2). We then measured ROS production in response to PMA stimulus. CFTR KO MDMs demonstrated a significant reduction in max endpoint ROS production in response to PMA (Figure 3B), also similar to our findings in primary CF MDMs (4). ROS production was also higher in non-CF MDMs at initial recording with continued separation over time as shown in the representative time course (Figure 3B). As a result of the deficit in ROS production, we measured expression of p47^*phox*^, a critical component necessary for NADPH oxidase activation and subsequent ROS generation. Total p47^*phox*^ expression was high, and phosphorylated p47^*phox*^ was low in response to PMA in CFTR KO MDMs, in contrast to non-CF MDMs (Figure 3C). We then measured cytosolic and membrane-bound fractions of phosphorylated p47^phox^ as recruitment of phosphorylated p47^*phox*^ to the plasma membrane is necessary for functional NADPH oxidase assembly. CFTR KO MDMs showed decreased phosphorylated p47^phox^ in the membrane-bound fraction (Figures 3C–E), again similar to prior findings in human CF MDMs (4). Together, these results suggest that loss of CFTR results in reduced macrophage stability and altered assembly of the NADPH oxidase complex, with resulting decreased ROS production.

### CFTR KO Reduces Bacterial Phagocytosis and Killing

**Figure 4:**
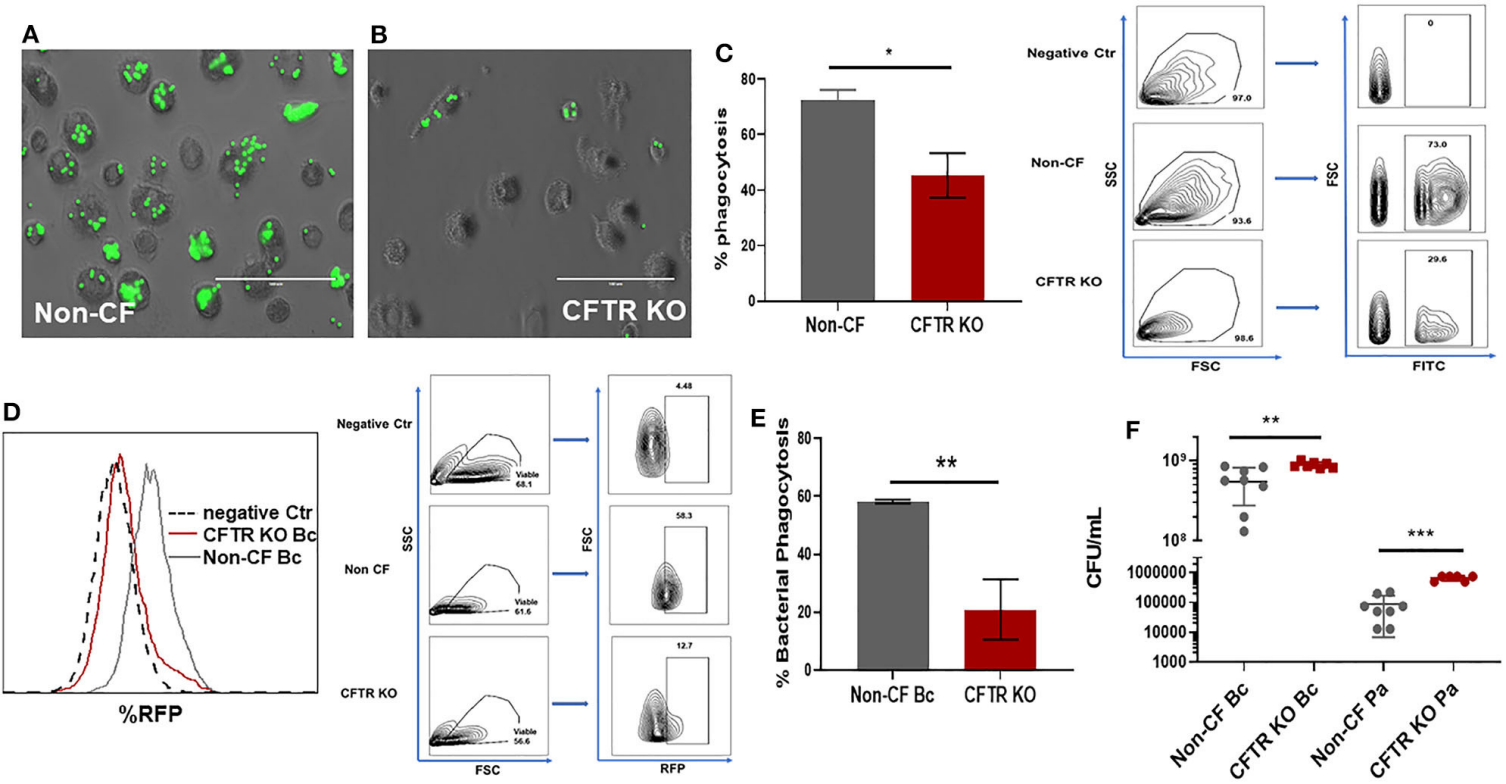
CFTR KO reduces bacterial phagocytosis and killing. Phagocytosis of FITC-labeled beads for **(A)** non-CF and **(B)** CFTR KO MDMs, 100 μm scale bar. Non-phagocytosed beads are washed away before imaging. **(C)** Summed % phagocytosis and representative FACS gating strategy for **(A,B)**, *n* = 3 donors, mean ± SD. Gating on FSC, SSC, and FITC. Phagocytosis shown as percent of cells with internalized beads. **(D)** Representative flow cytometry histogram and FACS gating strategy for bacterial phagocytosis assay for non-CF and CFTR KO MDMs. Histogram with overlay difference in uptake of RFP-labeled *B. cenocepacia* (Bc). The dashed black line (negative Ctr) were non-CF MDMs without infection. FACS gating on FSC, SSC, and RFP. **(E)** Summed %phagocytosis of RFP-expressing *B. cenocepacia, n* = 3/group, MOI 50, mean ± SD. **(F)** CFU assay in non-CF and CFTR KO MDMs infected with *B. cenocepacia* (Bc) or *P. aeruginosa* (Pa), log scale, *n* = 6–8 donors, mean ± SD. *p < 0.05, **p < 0.01, ***p < 0.001, via unpaired *t*-test.

Deficits in bacterial phagocytosis by CF monocytes and macrophages have been reported by numerous groups (2, 18–20, 28). However, a recent study demonstrated no difference between CF and non-CF monocyte phagocytosis of opsonized *Escherichia coli* (29), although this study was done in whole blood. To clarify these discrepancies, we measured phagocytosis in non-CF and CFTR KO MDMs with opsonized beads. CFTR KO was associated with a significant decrease in phagocytosis of beads compared to non-CF MDMs (Figures 4A–C). We also measured phagocytosis of RFP-labeled *B. cenocepacia*. CFTR KO was associated with an approximately 40% reduction in bacterial phagocytosis compared to non-CF (Figures 4D,E). Both bead and bacterial results were comparable to our prior data in primary human CF MDMs (2). To determine if these deficits in phagocytosis were associated with decreased killing of bacteria in the CFTR KO model, we measured killing of clinical isolates of three important CF pathogens, *Burkholderia cenocepacia*, and *Pseudomonas aeruginosa*. CFTR KO was associated with significantly higher bacterial burden of both *B. cenocepacia* and *P. aeruginosa* (Figure 4F, log scale) compared to non-CF MDMs. Together, these results demonstrate that CFTR is necessary for macrophage phagocytosis and subsequent killing of bacteria.

### CFTR KO Is Not Associated With Increased Cytokine Production

**Figure 5:**
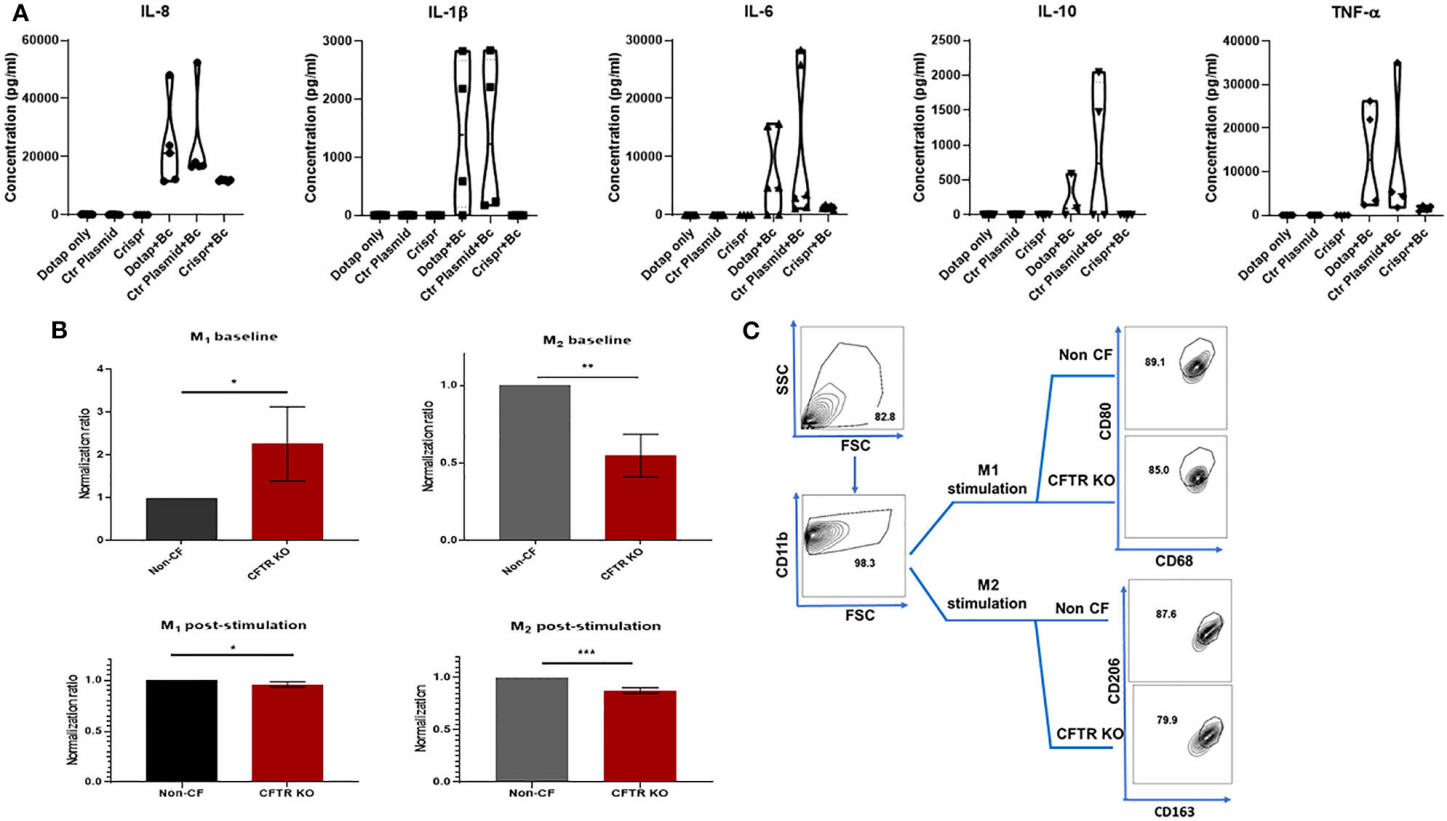
CFTR KO is associated with decreased cytokine and polarization responses. **(A)** Violin plots for IL-8, IL-1β, IL-6, IL-10, and TNF-α cytokine production. Cytokines were measured in macrophage supernatants at baseline for three conditions (DOTAP medium alone, transfection with an empty vector [Ctr plasmid], or CFTR KO [Crispr]). Cytokines were also measured during infection with *B. cenocepacia* (Bc) for the same three conditions as occurred at baseline. Significance determined via one-way ANOVA for baseline or infected conditions. *n* = 4–7 per group. **(B)** M1 (%CD68+ CD11b+ CD80+) and M2 (%CD11b+ CD163+ CD206+) polarization of non-CF and CFTR KO MDMs without a polarizing stimulus (top, baseline), and after 48 h exposure to a polarizing stimulus (bottom, post-stimulation). Data were normalized to non-CF MDMs. *n* = 4, mean ± SD. *p < 0.05, **p < 0.01, ***p < 0.001, via unpaired *t*-test. **(C)** FACS gating strategy for 5B post-stimulation studies. M1 & M2 MDM subsets were sequentially gated on FSC/SSC, CD11b, and CD68^+^CD80^+^ (M1) or CD163^+^CD206^+^(M2). Gating for M1 and M2 baseline from 5B was the same except for lack of M1/M2 stimulus.

CF is characterized by heightened inflammation, and in particular, increased macrophage cytokine production during infection or other stimuli (2, 6, 22, 30–34). To determine if CFTR KO alters macrophage cytokine production, we measured production of 5 cytokines (IL-8, IL-1β, IL-6, IL-10, and TNF-α) at baseline and in response to infection with *B. cenocepacia*. At baseline there were no differences in cytokine production between non-CF MDMs in media alone (DOTAP), when transfected with an empty vector (Ctr plasmid), or during CFTR KO (Crispr) (Figure 5A). There were significant reductions in IL-8, IL-6, and TNF-α production during infection in CFTR KO MDMs when compared to media alone or empty vector transfection (Figure 5A). There was also no significant difference in IL-1β or IL-10 production between CFTR KO MDMs compared to media or empty vector, although all CFTR KO MDMs had less IL-1β production compared to empty vector alone. These results suggest that CFTR KO alone does not recapitulate the increased cytokine production observed in human CF MDMs.

### CFTR KO Has a Minimal Effect on Macrophage Polarization

Macrophages can exist in different polarized states such as M1 (inflammatory) or M2 (anti-inflammatory/reparative) subsets. Previously reported alterations in CF macrophage polarization phenotypes differ depending on individual factors such as age or medication use, experimental conditions, and use of monocytes or differentiated macrophages (2, 20, 35–37). Because of these prior discrepancies and the unexpected cytokine findings from Figure 5A, we measured changes in macrophage polarization in response to CFTR KO. In unstimulated states, CFTR KO was associated with increased M1 MDMs and decreased M2 MDMs compared to non-CF MDMs (Figures 5B,C). However, in response to M1 or M2a polarizing stimuli, there were small but significantly decreased proportions of both M1 and M2a MDMs after CFTR KO compared to non-CF MDMs (Figures 5B,C). These findings correlate with the decreased cytokine responses from CFTR KO MDMs in Figure 5A.

## Discussion

CFTR dysfunction leads to the production of dehydrated mucus, impaired immunity, and subsequent chronic infections that cause progressive lung damage. We now recognize the role of underlying deficits in macrophage and overall innate immune responses that allow bacteria to establish in the CF lung (5, 12, 15, 32, 38–43). It remains unclear whether deficits in CF immune function are a result of intrinsic defects in CFTR (2, 32, 44), a result of the inflammatory CF lung milieu [reviewed in (21, 22)], or more likely a combination of both factors. Through generation of a CFTR KO model in human macrophages we demonstrated several aspects of macrophage dysfunction that are dependent on the presence of CFTR. Conversely, other aberrations observed in primary macrophages, such as increased cytokine production, may be a result of local environmental influences or epigenetic changes over time (22). These findings have implications for future approaches to improve immune responses to infections in CF.

In this study we found that CFTR KO resulted in an increased propensity for macrophages to undergo apoptosis. This finding is consistent with our previous study of CF macrophage (CFTR class I/II/III variants) responses to CFTR modulators, where primary human CF MDMs had increased apoptotic rates that could be reduced with ivacaftor treatment (2). However, primary CF sputum macrophages demonstrate low rates of apoptosis, despite a high likelihood of being macrophages recruited from the peripheral blood (45). These differences in apoptosis may reflect macrophage adaptations to a more stressful lung environment, or may simply be indicative of relative differences in apoptosis between macrophages and other immune cell populations (e.g., neutrophils) when studied simultaneously. Although our findings are consistent with increased apoptosis in alveolar macrophages that have decreased CFTR after treatment with CFTR specific small interfering RNA (CFTR-siRNA) (46), we believe that adaptation to the CF lung likely influences CF macrophage responses. Future studies need to examine if therapies that decrease CF macrophage apoptosis allow for improved functional capabilities, whether increased CF macrophage apoptosis contributes to the accumulation of inflammatory cellular debris in the lung, and how CF macrophage apoptosis influences macrophage extracellular trap formation that may also be involved in pathogen clearance or sterile inflammation.

While increased apoptosis could have a variety of impacts upon CF macrophage responses, we also found that CFTR KO decreased two important aspects of macrophage host defenses, phagocytosis and ROS production. These decreased defense mechanisms likely create a two-pronged insult that contributes to the increased bacterial loads observed with CFTR KO. CF macrophages are first unable to effectively phagocytose bacteria, and if successful at phagocytosis are subsequently unable to mount effective intracellular killing responses. CF macrophages have been previously shown to have several deficits related to phagocytosis, including deregulated transient receptor potential cation channel subfamily V member 2 (TRPV2)-mediated calcium influx, (19) reduced Ezrin-mediated toll-like receptor-4 (TLR4) signaling (47), and reduced complement-mediated phagocytosis (28). Additionally, ROS responses are altered in both human CF macrophages (4) and CF neutrophils (17). We are currently investigating the mechanism whereby CFTR dysfunction results in defective NADPH oxidase assembly (as shown by decreased phosphorylation of cytosolic components), and whether this deficit is dependent on phagocytosis-mediated signaling.

In contrast to most findings in this study, changes in cytokine production with CFTR KO did not reflect alterations previously observed in human CF macrophages (2, 6, 21, 22). Surprisingly, CFTR KO was associated with decreased production of several inflammatory cytokines in response to infection. Decreased cytokine production was in contrast to a prior study of siRNA-silencing of CFTR in alveolar macrophages (46), although the authors only examined basal IL-8 production and not responses to infection. Our findings may be related to the small but significant decrease in the ability of CFTR KO macrophages to respond to a pro-inflammatory M1 stimulus, despite a higher proportion of M1 macrophages after CFTR KO in the absence of infection. The observed deficits in CFTR KO macrophage responses to M1 or M2 polarization stimuli are similar to what we observed in primary CF macrophages (2), as well as decreased CF macrophage M2 polarization responses shown by other groups (20). Other studies have shown that medications such as azithromycin can alter CF macrophage polarization (36), but our studies were performed in the absence of any *in vitro* medication exposures. It is also possible that other laboratory conditions influence the polarization status such as the type of media, serum, or polarizing stimuli (48). However, because we did not have an increase in M2 polarized macrophages after CFTR KO, we do not think our conditions would be related to decreased inflammatory cytokine production in CFTR KO only. We suggest that all the findings combined support the assertion that CF macrophages are not inherently hyper-inflammatory, but are metabolically re-programmed to a hyper-inflammatory state in CF as originally postulated by Wilson and Fudenberg (49) and supported by recent studies (31). The concept of induced innate immune memory (50) also needs further investigation in CF and could contribute to hyper-inflammatory macrophage signaling. However, early and sustained use of new CFTR modulators may help reverse potential alterations in macrophage inflammatory responses that adversely impact CF outcomes. Prior studies of first-generation CFTR modulators have not shown sustained reductions in sputum inflammatory markers (51, 52). New investigations into early immune responses in CF are the focus of several ongoing studies around the globe.

This study was limited to the use of MDMs and did not include alveolar, sputum, or tissue-resident macrophages, which may respond differently to CFTR KO based on distinct transcriptional profiles in CF (53). Alveolar macrophages have become more difficult to obtain since the recent release of elexacaftor/tezacaftor/ivacaftor, which has decreased rates of expectorated sputum and clinical indications for flexible bronchoscopies. MDMs are recruited to sites throughout the body (including the lungs) and remain an easily accessible source of CF macrophages, lending utility to the current model for future studies in the era of highly effective CFTR modulator therapy. The CFTR KO model could also be used to test new therapeutic strategies in patients with class I CFTR variants, as this model closely resembles a clinical state where no CFTR protein is expressed. However, it will remain important for future studies to compare the model to primary patient macrophages of different CFTR variants as well as use CFTR channel inhibition with chemical inhibitors to look at the role of ion signaling. It is also possible that the cytokine differences observed in our KO model compared to primary human CF macrophages are an inherent limitation of the KO model. Although our control samples with transfection media alone or empty vehicle did not demonstrate altered cytokine responses, these studies can be further validated in future studies looking at long-term primary macrophage responses to highly effective CFTR modulators.

In summary, we developed a CRISPR-Cas9 CFTR KO model to study the impact of CFTR on macrophage function. CFTR KO recapitulated alterations in primary CF macrophage functions including apoptosis, phagocytosis, ROS production, and bacterial killing. CFTR KO was not associated with increased inflammatory cytokine production. This study suggests that many aspects of CF macrophage dysfunction are CFTR-dependent, which is important as we develop new approaches to regulate immune responses to infection in CF.

## Data Availability Statement

The raw data supporting the conclusions of this article will be made available by the authors, without undue reservation.

## Ethics Statement

The studies involving human participants were reviewed and approved by Nationwide Children's Hospital IRB (IRB16-01020). Written informed consent to participate in this study was provided by the participants' legal guardian/next of kin.

## Author Contributions

SZ performed scientific experiments, contributed to study design and analysis, and edited the manuscript. CS assisted with scientific experiments and edited the manuscript. BW assisted with experiments and edited the manuscript. HP collected patient samples and edited the manuscript. XH, WL, and YD assisted in implementation and optimization of the CRISPR-Cas9 KO model and edited the manuscript. BK designed the experiments, recruited participants, analyzed data, and wrote the manuscript. All authors contributed to the article and approved the submitted version.

## Conflict of Interest

BK has previously served on the US CF Advisory Board for Vertex Pharmaceuticals to provide advice on clinical trial development. The remaining authors declare that the research was conducted in the absence of any commercial or financial relationships that could be construed as a potential conflict of interest.

## Abbreviations

APC: allophycocyanine
APC/Cy7: allophycocyanin cyanine 7 tandem conjugate
CFTRinh172: CFTR inhibitor 172
CRISPR-Cas9: clustered regularly interspaced short palindromic repeats associated protein 9
cDNA: complementary DNA
CF: cystic fibrosis
CFTR: cystic fibrosis transmembrane conductance regulator
CFU: Colony forming unit
DCF: 2′,7′-dichlorofluorescein
EDTA: Ethylenediaminetetraacetic acid
ET12: Edinburgh-Toronto epidemic lineage strain
FACS: fluorescence-activated cell sorting
HEPES: 4-(2-hydroxyethyl)-1-piperazineethanesulfonic acid
HBS: HEPES buffered saline
IBMX: 3-isobutyl-1-methylxanthine
KO: knockout
LPS: lipopolysaccharide
LB: Luria-Bertani
MDMs: monocyte-derived macrophages
MOI: multiplicity of infection
DOTAP: N-[1-(2,3-Dioleoyloxy)propyl]-N,N,N-trimethylammonium methyl-sulfate
MQAE: N-Ethoxycarbonylmethyl-6-Methoxyquinolinium Bromide
NADPH: nicotinamide adenine dinucleotide phosphate
PE: phycoerythrin conjugate
PECy7: phycoerythrin cyanine 7 tandem conjugate
PKC: protein-kinase C
PBS: phosphate buffered saline
PMA: phorbol 12-myristate 13-acetate
ROS: reactive oxygen species
RFUs: relative fluorescent units
rhIFN-γ: recombinant human interferon gamma
RPMI: Roswell Park Memorial Institute
qRT-PCR: semi-quantitative reverse transcription polymerase chain reaction
siRNA: small-interfering RNA
TLR4: toll-like receptor-4
TRPV2: transient receptor potential cation channel subfamily V member 2.
