# Supplementary figures and images for: Consequences of CRISPR-Cas9-Mediated CFTR Knockout in Human Macrophages
Source: Front Immunol. 2020 Aug 18;11:1871. doi: 10.3389/fimmu.2020.01871 (PMC7461958; doi:10.3389/fimmu.2020.01871)

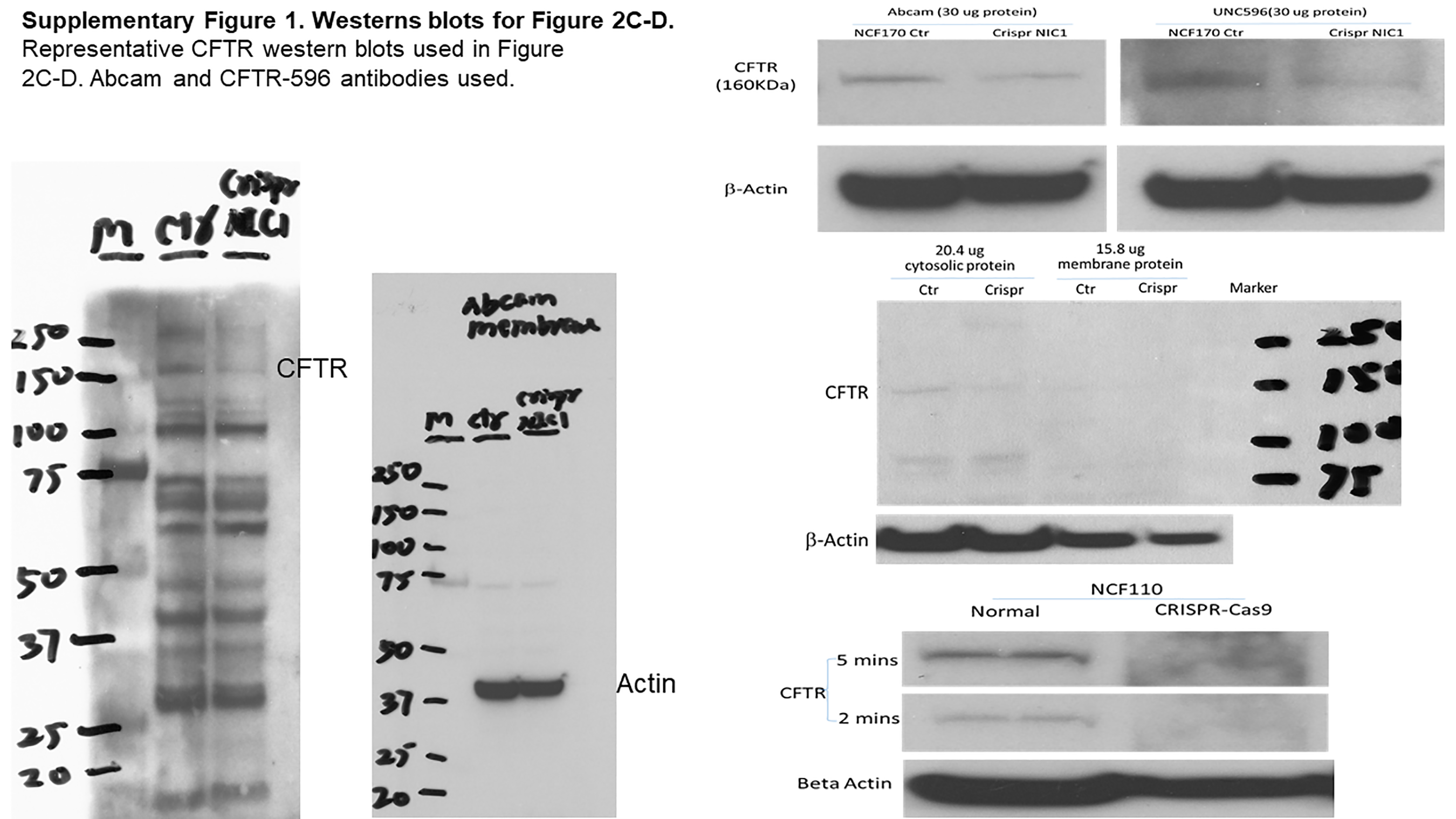

Supplement: Supplementary file 1 [file Image_1.TIF]
